# Bone marrow cell migration to the heart in a chimeric mouse model of acute chagasic disease

**DOI:** 10.1590/0074-02760160526

**Published:** 2017-08

**Authors:** Camila Iansen Irion, Bruno Diaz Paredes, Guilherme Visconde Brasil, Sandro Torrentes da Cunha, Luis Felipe Paula, Alysson Roncally Carvalho, Antonio Carlos Campos de Carvalho, Adriana Bastos Carvalho, Regina Coeli dos Santos Goldenberg

**Affiliations:** Universidade Federal do Rio de Janeiro, Rio de Janeiro, RJ, Brasil

**Keywords:** Chagas disease, chimeric mice, heart, bone marrow

## Abstract

**BACKGROUND:**

Chagas disease is a public health problem caused by infection with the protozoan *Trypanosoma cruzi.* There is currently no effective therapy for Chagas disease. Although there is some evidence for the beneficial effect of bone marrow-derived cells in chagasic disease, the mechanisms underlying their effects in the heart are unknown. Reports have suggested that bone marrow cells are recruited to the chagasic heart; however, studies using chimeric mouse models of chagasic cardiomyopathy are rare.

**OBJECTIVES:**

The aim of this study was to investigate the migration of bone marrow cells to the heart after *T. cruzi* infection in a model of chagasic disease in chimeric mice.

**METHODS:**

To obtain chimerical mice, wild-type (WT) C57BL6 mice were exposed to full body irradiation (7 Gy), causing bone marrow ablation. Then, bone marrow cells from green fluorescent protein (GFP)-transgenic mice were infused into the mice. Graft effectiveness was confirmed by flow cytometry. Experimental mice were divided into four groups: (i) infected chimeric (iChim) mice; (ii) infected WT (iWT) mice, both of which received 3 × 10^4^ trypomastigotes of the Brazil strain; (iii) non-infected chimeric (Chim) mice; and (iv) non-infected WT mice.

**FINDINGS:**

At one-month post-infection, iChim and iWT mice showed first degree atrioventricular block with decreased heart rate and treadmill exercise parameters compared to those in the non-infected groups.

**MAIN CONCLUSIONS:**

iChim mice showed an increase in parasitaemia, myocarditis, and the presence of amastigote nests in the heart tissue compared to iWT mice. Flow cytometry analysis did not detect haematopoietic progenitor cells in the hearts of infected mice. Furthermore, GFP+ cardiomyocytes were not detected in the tissues of chimeric mice.

Chagas disease (CD), caused by the protozoan *Trypanosoma cruzi*, is an important health problem in Central and South America (Rassi Jr et al. 2010). Reports from World Health Organization (WHO 2016) estimate that 6-7 million people are infected worldwide. Although a decrease in incidence has been observed in endemic countries, CD is increasing in other places, such as the United States of America, Canada, Japan, Australia, and Europe due to migratory movement (Rassi Jr et al. 2010). Heart impairment is the most severe and frequent manifestation of this disease. During the acute phase, infection with *T. cruzi* leads to high parasitaemia and tissue parasitism, which can result in an exacerbated immune response in the heart (Gutierrez et al. 2009), including infiltration of CD4+ and CD8+ T lymphocytes and macrophages, with a predominance of CD8+ T cells (Machado et al. 2005). Depletion of lymphocytes in murine models results in high susceptibility to *T. cruzi*, showing that CD4+ and CD8+ T cells are essential for parasite control and host survival (Tarleton et al. 1996). In the chronic phase, parasites are rarely found in the blood. However, small and progressive inflammatory foci continue to develop for years, culminating in the destruction of cardiomyocytes, formation of fibrosis, and the development of dilated cardiomyopathy (Gutierrez et al. 2009) in approximately 30% of infected individuals, which can progress to congestive heart failure (Soares et al. 2004). At this stage, heart transplantation is often the only therapeutic option. However, cell therapy has emerged as a promising alternative treatment. Recent advances have shown the beneficial effects of progenitor cells from bone marrow (BM) in different tissues (Epperly et al. 2003, Abedi et al. 2007), including the heart (Orlic et al. 2001, Yoshioka et al. 2005, Goldenberg et al. 2008, Jasmin et al. 2012, Souza et al. 2014). In a mouse model of chronic chagasic cardiomyopathy, mononuclear cells from BM decreased inflammatory infiltrates and fibrosis (Soares et al. 2004) and prevented dilatation of the right ventricle (Goldenberg et al. 2008). In an acute model, mesenchymal stem cells from BM also promoted a reduction of right ventricular dilatation (Jasmin et al. 2012).

Although there is strong evidence for a beneficial effect of BM-derived cells in chagasic and other cardiomyopathies, the mechanisms underlying the effects of BM cells in the heart are still unknown. Reports have shown that BM cells are recruited to repair the heart in a model of myocardial infarction (Orlic et al. 2001, Fazel et al. 2006). Transplantation of BM expressing green fluorescent protein (GFP) into irradiated mice has been used to screen and study BM cell participation in tissue repair in different organs, including the heart (Epperly et al. 2003, Abedi et al. 2007, Fujita et al. 2007, Souza et al. 2014). Chimeric mice have been used in myocardial infarction models to show the beneficial effects of BM CD117+ cells (Fazel et al. 2006) and granulocyte colony-stimulating factor (G-CSF) (Fujita et al. 2007) on cardiac tissue. However, there are few studies of CD using chimeric mouse models (Soares & dos Santos 2009, Souza et al. 2014). In this study, we investigated BM cell migration to the heart after *T. cruzi* infection in an experimental model of acute CD in chimeric mice and compared parasitaemia, myocarditis, parasite nests, and cardiac function in infected wild-type (WT) and chimeric mice.

## MATERIALS AND METHODS

*Animals* - Male and female C57Bl/6 mice and transgenic green fluorescent protein-expressing mice [C57Bl/6-Tg(CAG-EGFP) C14-Y01-FM131O (GFP mice)] were donated by Dr Masaru Okabe (Okabe et al. 1997) and housed in our animal facility at the Carlos Chagas Filho Biophysics Institute (UFRJ, Rio de Janeiro, Brazil). The facility is temperature controlled (23ºC), with a 12-12h light-dark cycle and free access to water and standard chow. The investigation was carried out in accordance with the Guide for the Care and Use of Laboratory Animals [DHHS publication no. (NIH) 85-23, revised in 1996, Office of Science and Health Reports, Bethesda, MD 20892, USA] and all animal protocols were approved by the Ethics Committee on the Use of Animals in Scientific Experimentation (Health Science Centre of the Federal University of Rio de Janeiro), under protocol number 163/13.

*Generation of chimeric mice* - To generate chimeric mice, 34 C57Bl/6 mice (weighing approximately 25 g) were exposed to whole body radiation (7 Gy) in a linear accelerator (VarianClinac 2100 CD) used for radiotherapy, resulting in BM ablation as previously described (Paredes et al. 2012). BM cells were harvested from the femurs and tibiae of GFP transgenic mice at two months of age by centrifugation (1500 × *g* for 3 min). To isolate mononuclear cells, Bone Marrow Cells BMCs were suspended in Dulbecco’s Modified Eagle’s Medium (DMEM; LGC Biotecnologia) and layered on Histopaque (Histopaque^®^-1083, Sigma-Aldrich). The tubes were centrifuged at 400 × *g* for 30 min. The ring containing the mononuclear cells was collected and washed; the cells were counted and cell viability was determined by Trypan blue exclusion. Mononuclear cells were diluted in sterile phosphate buffered saline and injected (at 2 × 10^6^ cells/200 µL) through an intracavitary cardiac route guided by echocardiography into irradiated WT mice approximately 4 h after irradiation.

*Engraftment evaluation* - The percentage of BM engraftment was determined in irradiated/transplanted mice 21 days after the procedure. Blood samples from WT C57Bl/6 and transgenic GFP+ mice were used as negative and positive controls, respectively. Peripheral blood (PB) samples (20 µL) collected from the tail vein were incubated with 200 µL of erythrocyte-lysing solution (BD FACS Lysing Solution, BD Biosciences) for 15 min at room temperature, washed with PBS, centrifuged at 300 × *g* for 3 min, and then suspended in PBS (300 μL) for flow cytometry analysis (BD FACSAria IIu). Chimeric mice with greater than 70% engraftment of GFP-positive cells in PB were included in the study.

*Preparation of antigens (lysed parasites)* - To avoid high mortality of irradiated mice after *T. cruzi* infection, the mice were injected with lysed parasites before infection. To obtain lysed parasites, a monkey fibroblast cell line (LLC-MK2) was infected with *T. cruzi* trypomastigotes (Brazil strain; at a 1:1 parasite:cell ratio). The cells were maintained in DMEM supplemented with 2% foetal bovine serum (FBS) at 37ºC with 5% CO_2_. From day five to eight, parasites were harvested from the culture supernatant, centrifuged (1200 × *g* for 15 min), resuspended in 1 mL of PBS, and counted in a Neubauer chamber. A suspension containing 10^6^ parasites/mL in PBS was lysed by three freeze-thaw cycles in a water bath at 37ºC and liquid nitrogen for 5 min each. A complete loss of viability was verified by the absence of parasite bodies via light microscopy. The lysed parasite suspension (200 µL) was injected by the intraperitoneal (IP) route 22 days before *T. cruzi* inoculation.

*T. cruzi infection and screening* - Infected groups of mice (males and females, aged 4-5 months) were inoculated by IP injection with 3 × 10^4^ bloodstream trypomastigotes of the Brazil strain. Five days post infection (dpi), parasitaemia was assessed by counting the number of trypomastigotes in PB diluted in 0.85% ammonium chloride buffer in a Neubauer chamber, three times a week. Data are expressed as the number of trypomastigotes per millilitre of blood. Mortality was assessed daily.

**Fig. 1:**
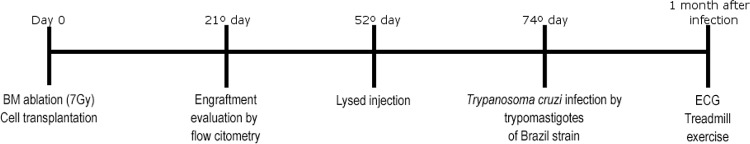
: experimental design of chimeric generation and *Trypanosoma cruzi* infection. C57Bl/6 mice were exposed to whole body radiation (7 Gy; Day 0) in a linear accelerator (VarianClinac 2100 CD). Approximately 4 h after irradiation, irradiated mice were administered 2 × 106 bone marrow (BM) mononuclear cells through an intracavitary cardiac route guided by echocardiography. Engraftment was evaluated 21 days after irradiation by flow cytometry. Fifty-two days after irradiation, chimeric mice received an antigen preparation (a suspension containing 106 lysed *T. cruzi* parasites), and 22 days later (at 74 days after irradiation), animals were infected with 3 × 104*T. cruzi* trypomastigotes (Brazil strain) by the intraperitoneal route.

*Experimental groups* - Animals were divided into four groups: (i) the iChim group (n = 17) and (ii) the iWT group (n = 17), which were composed of chimeric and WT C57Bl/6 mice, respectively, that received *T. cruzi* lysate (ip) at 52 days-post irradiation (dpir) and were infected with *T. cruzi* 22 days after lysate injection (74 dpir), and age-matched non-infected controls in the (iii) Chim group (n = 13) and (iv) WT group (n = 14). An experimental overview of chimera generation and infection is shown in Fig. 1.

*Cardiac performance* - Cardiac performance was evaluated by electrocardiogram (ECG) and treadmill exercise before infection and one month after infection. ECG recordings were obtained in conscious animals, using an electrocardiographic unit (PowerLab/400; AD Instruments), and the following parameters were analysed: the presence of arrhythmias, PR interval, QT interval, Bazett-corrected QT (QTc = QT/√iRR), and heart rate (HR = 60/iRR). Treadmill exercise was performed using a motor-driven treadmill (EP 131; Insight Instruments, Ribeirão Preto, Brazil) at a 10º inclination, according to the protocol of exhaustion adapted from Bayat et al. (2002). Briefly, the mice were familiarised with the treadmill by running at a speed of 0.1 m/s for 5 min before beginning exercise. Then, the animals were exercised at five different velocities (0.1, 0.2, 0.3, 0.4, and 0.5 m/s) with increasing velocity. Each mouse ran until fatigue, defined as the point when the animal remained more for than 10 s on the electrified grid present at the end of each stall. Travelled distance (metres) and time (minutes) until fatigue were used as indexes of exercise tolerance.

*Histopathological and morphometric analysis* - Hearts from all groups were arrested in diastole, at an average of 35 days, washed with PBS, and sectioned transversally in two segments (apex and base). Heart sections were fixed in 4% buffered paraformaldehyde for 24-48 h, embedded in paraffin, sectioned (4 µM), and stained with hematoxylin-eosin (H&E) to evaluate the intensity of inflammation and presence of amastigote nests. Tissue sections were analysed by light microscopy, and images were digitalised using a colour digital camera (Spot Flex) attached to an epifluorescence Axiovert 100 microscope (Zeiss, Germany). For morphometric analyses, each section (for a total of 30 microscope fields, from the left and right ventricles) was examined for evidence of mononuclear and polymorphonuclear cellular infiltration and amastigote nests. Inflammation was determined by counting the number of inflammatory foci (1 focus = seven or more inflammatory cells) within a specific area (5 μM^2^). The presence of amastigote nests was determined in the same way, by counting the number in 30 fields. The images were analysed using Image-Pro Plus, version 7.0 (Media Cybernetics, Bethesda, MD, USA).

*Immunophenotyping by flow cytometry* - Immunophenotyping of the BM and hearts of mice from the infected [iChim (n = 3) and iWT (n = 3)] and control [Chim (n = 3) and WT (n = 3)] groups were performed at 40 days post-infection, using a BD FACSAria IIu (BD Biosciences), and the data were analysed using FlowJo software (Treestar, USA). DAPI (500 ng/mL) was used to exclude dead cells, and isotypes (IgG2b-PE and IgG2a-PE-Cy7) were used as negative controls.

*BM* - Total BM was harvested as described above and incubated with Fc block (anti-CD16/CD32; BD Biosciences) for 15 min at 4ºC. Then, the cells were incubated for 30 min at 4ºC with conjugated primary antibodies against hematopoietic progenitors (anti-CD45-PE-Cy7/anti-CD117-PE), washed in PBS, centrifuged (300 × *g* for 5 min), and resuspended in PBS (200 µL) containing 0.5% BSA.

*Hearts* - After perfusion with PBS at a constant flow of 1.1 mL/min for 20 min, each heart was harvested and subjected to enzymatic digestion with collagenase type II (Worthington^®^) solution (200 U/mL). The suspension was subjected to five cycles of digestion at 37ºC for 5 min each. Cell suspensions were filtered using a 100-µM strainer and centrifuged at 300 × *g* for 5 min. Pellets were resuspended in PBS containing 0.5% BSA. Cells were incubated with Fc block (anti-CD16/CD32; BD Biosciences) for 15 minutes at 4ºC and then with the following conjugated primary antibodies as markers of hematopoietic progenitors (anti-CD45-PE-Cy7/anti-CD117-PE) for 30 min at 4ºC. After antibody incubation, cells were washed in PBS (300 × *g* for 5 min) and resuspended in PBS (200 µL) containing 0.5% BSA. Green fluorescence was examined in cells having the correct size and granularity for cardiomyocytes in chimeric mice.

*Statistical analysis* - Normal distribution and variance homogeneity were verified using Shapiro Wilk and Bartlett tests, respectively. In cases where normality and variance homogeneity were confirmed, parametric tests were used and, the data are shown as the mean ± standard derivation. In cases where normality and variance homogeneity were not confirmed, nonparametric tests were used, and the data are shown as medians. To analyse parasitaemia time dependency, a general linear model was used. The parasitaemia peak and morphometric analysis were analysed by the Mann-Whitney U test. For electrocardiography and treadmill exercise, we used two-way analysis of variance (ANOVA) with Bonferroni’s post hoc test to correct for multiple comparisons. For flow cytometry, the Mann-Whitney U test was used for the Chimeric and WT groups. To evaluate survival, Kaplan-Meier curves were used, and analysed with the Gehan Breslow-Wilcoxon test. Results were considered significant at P values less than 0.05. MatLab R2010a, R, and GraphPad Prism 6 were used for statistical analyses.

## RESULTS

**Fig. 2:**
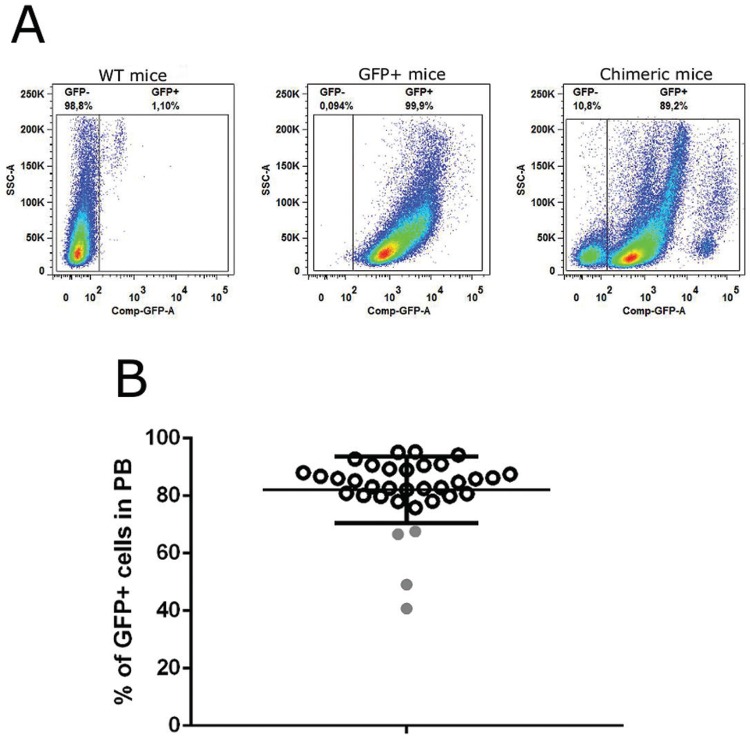
: engraftment evaluation of chimeric mice. (A) Dot-plot analysis of peripheral blood: Left: C57Bl/6 mice (negative control), middle: GFP+ mice (positive control), right: chimeric mice, 21 days after irradiation/bone marrow (BM) transplant showing two populations: GFP+ cells (right) and GFP- cells (left). (B) The percentage of BM engraftment in chimeric mice as analysed by flow cytometry. Of 34 transplanted mice, 30 showed greater than 75% engraftment (black circle) and were included in the study, and four showed less than 75% engraftment (grey circle) and were excluded. PB: peripheral blood; SSC-A: granularity; Comp-GFP-A: fluorescent intensity in FITC channel.

*Analyses of GFP+ cells derived from BM in PB to confirm engraftment* - To evaluate BM transplant efficacy in chimeric mice, PB was harvested and analysed by flow cytometry 21 days after transplantation of GFP BM cells. Quantification of GFP+ cells is shown in Fig. 2A. Of the 36 irradiated and transplanted mice, two (5.5%) died between transplant and engraftment evaluation. PB analysis showed that 30 out of 34 mice presented greater than 75% engraftment, with an average of 85.50% ± 5.33% CD45+/GFP+ cells, and they were included in the study (Fig. 2B).

**Fig. 3:**
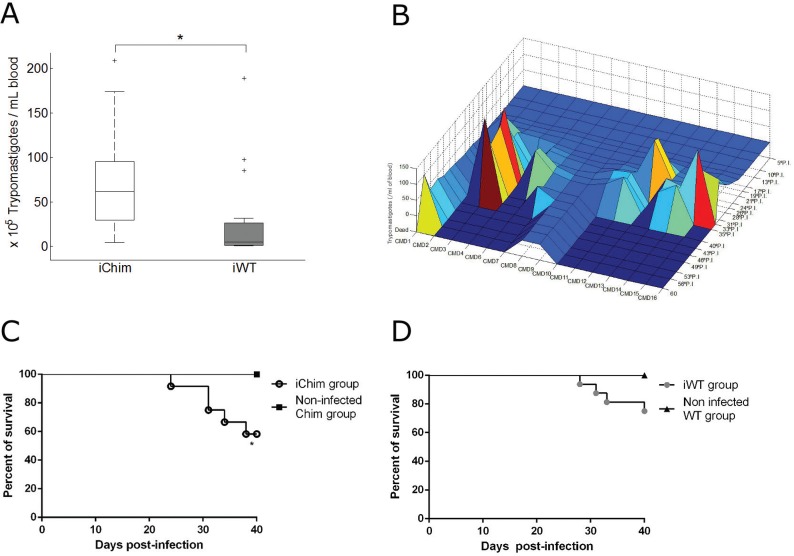
: parasitaemia and survival of chimeric and wild-type (WT) C57BL/6 mice infected with *Trypanosoma cruzi* (Brazil strain). (A) Median of the maximum number of circulating trypomastigotes from iChim (n = 15) and iWT (n = 17) groups. There is a significant difference between iChim [61.5 × 105 (29.25, 101.5)] and iWT [5.25 × 105 (1.13, 28.25) trypomastigotes/mL of blood] mice. (B) Parasitaemia over time showing marked variability in the number of trypomastigotes in the iChim group. Animals #5 and #17 were excluded from parasitaemia analyses because they died or were sacrificed at an early time point, which did not allow visualisation of peak parasitaemia; (C) Kaplan-Meier curve showing survival of infected and non-infected chimeric mice until 40 dpi. The iChim group showed a survival rate of 58.33%; (D) Kaplan-Meier curve showing the survival rate of infected and non-infected WT mice until 40 dpi. Mice in the iWT group showed a survival rate of 75%. None of the non-infected mice died. For parasitaemia, values were expressed as the median and inter quartile range (first-third quartiles) *p < 0.05 calculated by the Mann-Whitney U test.

*Course of infection: analysis of parasitaemia and survival* - As shown in Fig. 3A, assessment of parasitaemia levels beginning at 5 dpi revealed that the iChim group exhibited significantly higher parasitaemia than the iWT group [median (interquartile range); iChim, 61.5 × 10^5^ (29.25-101.5) vs. iWT, 5.25 × 10^5^ (1.13-28.25) trypomastigotes/mL of blood]. The graph shows that the maximum number of circulating trypomastigotes in each animal was time-independent. Fig. 3B shows the high variability in the number of trypomastigotes in the iChim group. Survival analyses showed a survival rate of 58.33% for mice in the iChim group, which was significantly different from that of the non-infected Chim control group (100% survival) until 40 dpi (Fig. 3C). The iWT group (Fig. 3D) showed a 75% survival rate, with no significant difference compared to the non-infected WT control group (100% survival). In addition, there was no significant difference in survival rates between the iChim and iWT groups.

*Cardiac performance - Electrocardiogram* - Both groups of infected mice showed disturbances in cardiac conduction by ECG, such as first degree AV block and abnormal cardiac rhythm one month after infection when compared to the ECGs of non-infected controls. As shown in Table, the iChim group exhibited a significantly higher PR interval (iPR) and a lower heart rate (HR) than the non-infected control Chim group. Moreover, these parameters were significantly different when the same animals were compared pre- and post-infection. Similarly, the iPR of the iWT group differed significantly when compared to that of the WT control group, and when the HR and iPR in the same animals were compared pre- and post-infection. For the QT interval and corrected QT interval, the infected groups did not show any significant differences (data not shown) when compared to values for the non-infected groups. Furthermore, there were no significant differences between the iChim and iWT groups.

**TABLE:** Electrocardiographic analysis of non-infected [Chim and wild-type (WT)] and infected groups (iChim and iWT) one month post-infection with trypomastigotes of Brazil strain

Groups/time	iPR (s)		HR (bpm)	
	
	Before infection	After infection	Before infection	After infection
Non infected chimeric (n = 13)	0.034 ± 0.002	0.034 ± 0.001	735.16 ± 30.66	754.02 ± 31.80
iChim (n = 9)	0.032 ± 0.002	0.040 ± 0.007^*a,b*^	736.41 ± 28.61	612.09 ± 94.58^*a,b*^
Non infected WT (n = 14)	0.032 ± 0.002	0.034 ± 0.001	744.26 ± 31.10	728.26 ± 36.85
iWT (n = 10)	0.033 ± 0.003	0.045 ± 0.010^*a,b*^	725.14 ± 57.94	618.14 ± 130.11^a^

a: difference before and after infection; b: difference between non-infected and infected groups one month after infection. Values are expressed as mean ± standard deviation (SD). a,b: p < 0.05 calculated by Two-way ANOVA with Bonferroni post-test. s: seconds; iPR: PR interval; HR: heart rate.

**Fig. 4:**
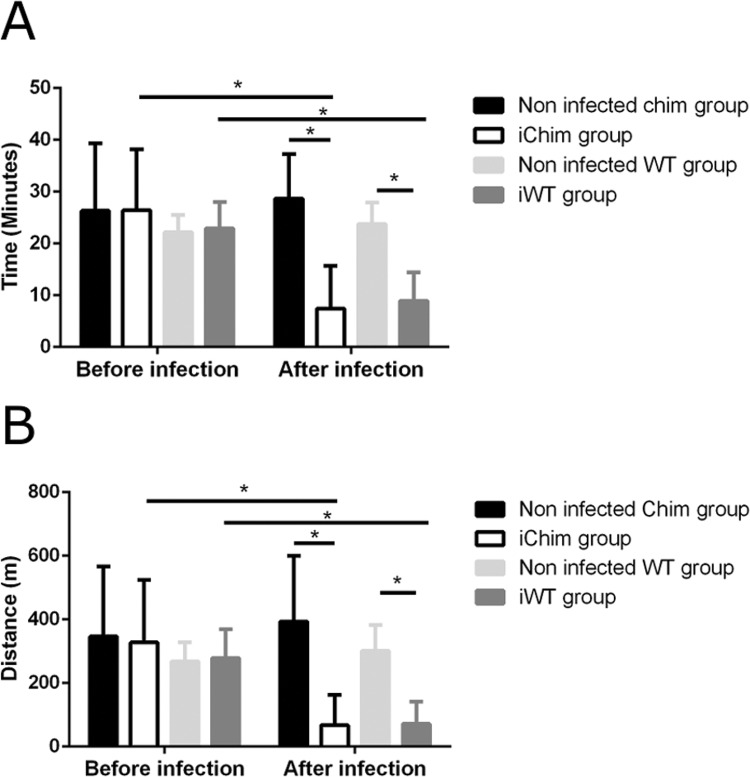
: cardiac performance evaluated by treadmill exercise testing. (A) Time travelled (in minutes) and (B) distance travelled (in metres) were calculated for each experimental group one month post-infection (Non-infected chimeric (Chim) group, n = 13; iChim group, n = 11; non-infected wild-type (WT) group, n = 14, and iWT group n = 13). M: metre. Values are expressed as mean ± SD. *p < 0.05, calculated by two-way ANOVA with Bonferroni post-test.

*Treadmill exercise* - An exercise test (Fig. 4) was used to assess cardiovascular performance in response to physical stress. We observed that infection with *T. cruzi* impaired exercise tolerance, as evidenced by significant decreases in time and distance travelled in the same animals before and after infection (Chimeric mice: before infection = 26.39 ± 11.79 vs. after infection = 7.43 ± 8.23 min, WT mice: before infection = 22.92 ± 5.11 vs. after infection = 8.95 ± 5.5 min; Chimeric mice: before infection = 327.72 ± 219.97 vs. after infection = 393.84 ± 205.57 metres, WT mice: before infection = 279.23 ± 89,84 vs. after infection = 71.92 ± 69.05 metres). In addition, there were significant differences in treadmill exercise time and distance when comparing infected groups to non-infected groups (non-infected Chim group = 28.67 ± 8.56 vs. iChim group = 7.43 ± 8.23 min, non-infected WT group = 23.76 ± 4.18 vs. iWT group = 8.95 ± 5.5 min; non-infected Chim group = 393.84 ± 205.57 vs. iChim group = 67.54 ± 94.96 metres, non-infected WT group = 301.93 ± 80.40 vs. iWT group 71.92 ± 69.05 metres). It is important to mention that no differences were observed between the iChim and iWT groups.

**Fig. 5:**
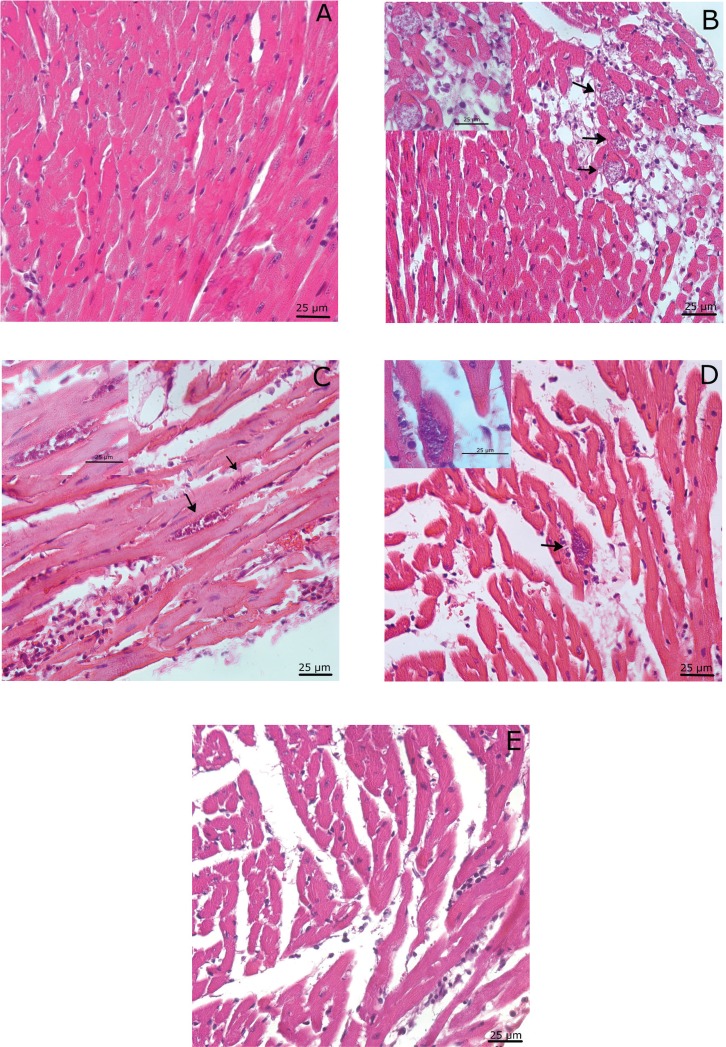
: histological analyses of the heart during acute infection. Sections of the heart stained with H&E from non-infected (A) and *Trypanosoma cruzi* (Brazil strain)-infected mice (B-E). Sections of the heart from mice in the iChim group (B-C) and infected wild-type (iWT) group (D-E) showing inflammatory foci and amastigote nests. In the controls groups (A) no tissue alteration was observed. Amastigote nests (black arrows) were observed in all infected groups: iChim group (4/5), WT-Lis group (1/5).

*Histopathologic and morphometric analyses of the heart* - The presence of inflammation and amastigote nests in the heart is a common manifestation in animals infected by *T. cruzi*; therefore, we compared heart sections of mice from the iChim and iWT groups during the acute phase of infection (average, 35 dpi). Fig. 5B-E shows histological analyses of hearts with inflammatory infiltrates, mainly mononuclear cells, and amastigote nests in the infected groups (Fig. 5B-D).

**Fig. 6:**
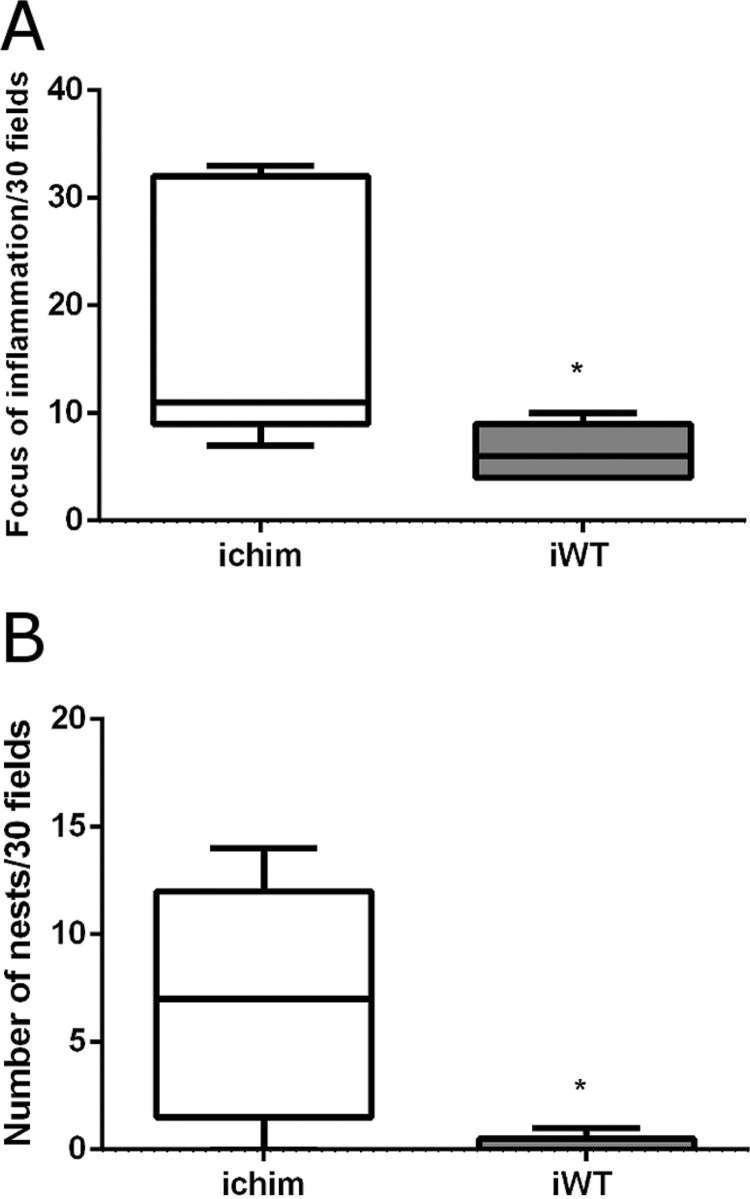
: morphometric analyses of the heart during acute infection Quantification of (A) inflammation foci and (B) amastigote nests from infected mice [Chimeric and wild-type (WT) groups]. Values are expressed as the median and inter quartile rage (first-third). *p < 0.05 calculated by Mann-Whitney U test.

No alterations were observed in the control groups (Fig. 5A). However, we observed that the hearts of mice in the iChim group had significantly higher numbers of inflammatory cells than the hearts of mice in the iWT group [iChim 11.0 (10-32) vs. iWT 6.0 (4-9), n = 5; Fig. 6A] and had higher numbers of amastigote nests than mice in the iWT group [iChim 7.0 (2.25-12), Fig. 6B vs. iWT 0 (0-0.5), Fig. 5D]. Data shown are the medians (interquartile range; first-third quartiles), and in the iWT group, the third quartile value of 0.5 indicates the presence of amastigote nests in one of the examined animals.

**Fig. 7:**
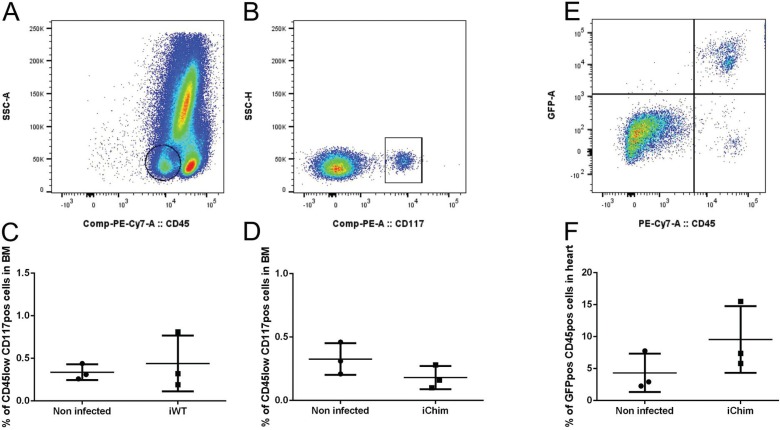
: immunophenotyping of bone marrow (BM) and heart from wild-type (WT) and chimeric groups by flow cytometry. Representative dot plots of CD45low (A) and CD117+ (B) cells in BM. The population of cells shown in B is derived from the circular gate shown in A. Quantification of hematopoietic progenitor cells (CD45low/CD117+) in WT (C) and chimeric (D) mice. (E) Representative dot plot of heart cells from chimeric mice showing the presence of CD45+/GFP+ and CD45+/GFP- cells. The second population is derived from the residual BM since the engraftment is not 100%. Rare events were observed in the CD45-/GFP+ gate (see Discussion). (F) Quantification of BM-derived CD45+/GFP+ cells.

*Analysis of cells in BM and BM-derived cells in the hearts of infected mice* - *WT group* - To determine if infection altered the number of hematopoietic progenitor cells (HPCs) in BM, we identified and quantified CD45low/CD117+ cells in BM (Fig. 7A-B). No difference was observed in the number of BM HPCs between non-infected and infected mice (Fig. 7C).

*Chimeric group* - The same analysis as described above was performed for non-infected and infected chimeric mice. No differences were observed between infected (iChim) and non-infected (Chim) groups regarding the number of HPCs in BM (Fig. 7D). Although GFP+ cells were detected in the heart (Fig. 7E), these cells were also CD45+, suggesting that they are inflammatory cells. There was a trend towards an increase in GFP+/CD45+ cells in the heart; however, this difference did not reach statistical significance (Fig. 7F) due to the low number of animals examined. Furthermore, no GFP+ cardiomyocytes (CD45-/GFP+) were detected by flow cytometry (Fig. 7E).

## DISCUSSION

In the present study, we detected and analysed GFP+ BM cells that migrated to the heart after *T. cruzi* infection in a chimeric mouse model of chagasic cardiomyopathy. Chimeric mice have been used to study the participation of BM cells in the regeneration of different tissues, such as skeletal muscle, the liver, and the heart (Epperly et al. 2003, Fazel et al. 2006, Abedi et al. 2007, Paredes et al. 2012, Souza et al. 2014). To develop this model, we followed the protocol used by Paredes et al. (2012), which had already been established in our laboratory, with some modifications. We used 7 Gy of radiation, and 2 × 10^6^ mononuclear cells were injected by an intracavitary cardiac route guided by echocardiography. The mean percentage of GFP+ cells in the PB 21 days post transplantation was greater than 75%. In our hands, BM GFP+ cell delivery through the intracavitary route was more efficient than other tested routes, such as the ocular route and through the tail. Although the engraftment was successful, the radiation exposure impaired the immune response. Park and Jo (2011) showed that irradiation of C57Bl/6 mice induced poor stimulation of the Th1 response, which is related to resistance in Chagas’ disease (Dosreis 1997). Similarly, Trischmann et al. (1982) reported that irradiation altered the immune responses of animals and consequently reduced survival. The authors performed a reciprocal transfer of BM cells between mice that are resistant and susceptible to *T. cruzi* infection; however, none of the chimeric mice survived infection.

To bypass the mortality of *T. cruzi* infection in BM-transplanted animals, we used parasite antigen stimulation, which has been described in the literature as a means to induce humoral and cellular immune responses in mice (Pereira et al. 2005, Bhatia & Garg 2008). Benznidazole has also been used to decrease the high mortality of chimeric mice after infection (Souza et al. 2014).

In our study, prior to infection, chimeric and WT mice were inoculated with a lysate of *T. cruzi* to stimulate the immune response and increase animal survival. Twenty-two days after lysate injection and 74 days after irradiation, mice were administered 3 × 10^4^ *T. cruzi* trypomastigotes (Brazil strain). It is important to mention that without lysate inoculation, all animals (n = 4) infected 74 days post BM transplantation died at 40 dpi (data not shown).

iChim mice developed higher parasitaemia and showed larger variability in parasitaemia when compared to mice in the iWT group. This variability has been described by Sogayar et al. (1993). In addition, Trischmann (1982) had demonstrated greater parasitaemia variability in chimeric animals (7.25 × 10^5^-366 × 10^5^ trypomastigotes/mL of blood). As expected, the higher parasitaemia detected in BM-transplanted animals resulted in greater inflammation and a higher number of amastigote nests in the hearts of chimeric mice. However, both infected chimeric and WT mice developed functional alterations compatible with chagasic cardiomyopathy at one month post-infection, as measured by ECG and treadmill exercise experiments.

Previous studies have demonstrated alterations in ECG and the parameters of exercise performance (Rocha et al. 2006, Macambira et al. 2009, Eickhoff et al. 2010). Eickhoff et al. (2010) evaluated different combinations of mouse strains (BALB/c, C57Bl/6, and C3H) and *T. cruzi* strains (Tulahuen, Brazil and Sylvio-X10/4). BALB/c mice infected with the Brazil and Tulahuen strains showed a prolonged QT interval. Furthermore, the authors also showed that C57Bl/6 mice infected with the Brazil strain showed no ECG changes. In contrast to the results of the study by Eickhoff (2010), in which the animals were infected with 5 × 10^3^ trypomastigotes, in our study, infected animals (Chim and WT) showed changes in cardiac electrical conduction, such as prolongation of the PR interval, at one month post-infection when compared to the values for non-infected controls. Moreover, changes in HR are common in CD and are associated with the presence of cardiac dysautonomy (Ribeiro et al. 2002), caused either by autonomic dysfunction or by a direct effect of the parasites on the Sino Atrial Node SAN. In our study, infected animals from both groups showed significant reductions in HR when compared to those of the control groups. Sympathetic innervation is deeply affected during the acute phase of the disease (Marin-Neto et al. 2007), thus explaining the observed reduction in HR.

As described by several authors, we observed a significant decrease in the distance and in the time travelled in infected mice when compared to control mice. Rocha et al. (2006) showed a low resistance to exercise with lower maximal oxygen uptake (VO2) in C57Bl/6 mice infected with the Colombian strain of *T. cruzi*. Using the combination of C57Bl/6 mice and the Colombian strain, Macambira et al. (2009) showed a significant reduction in the distance travelled and duration in exercise testing at 6 months after infection.

In our study, infected mice showed typical histopathological changes of acute chagasic myocarditis, with presence of amastigote nests in both ventricles as well as the presence of a focal inflammatory process, with a predominance of mononuclear cells. We also observed disorganisation of cardiac fibres and myocytolysis in some animals. Infected chimeric mice showed a greater number of amastigote nests and foci of inflammatory infiltrates when compared to those in iWT mice.

BM cells reportedly have the ability to migrate to injured organs where they might have beneficial effects (Orlic et al. 2001, Fazel et al. 2006). However, the mechanisms underlying these potential benefits are unknown. It has been proposed that HPCs may confer these beneficial effects either directly (Orlic et al. 2001) or indirectly (Yoshioka et al. 2005). We investigated whether *T. cruzi* infection induced changes in BM HPCs by flow cytometry. The number of CD45low/CD117+ HPCs in the BM of WT or Chim mice was not changed by infection, suggesting that HPCs in the BM are not a target of the parasite, at least during the acute phase of infection. Contrary to what has been found in an acute myocardial infarction model (Orlic et al. 2001), we could not detect the presence of BM-derived CD117+ cells in infected hearts, indicating that HPCs do not migrate to the heart during the acute phase of infection. Although CD45+/GFP+ cells were present in the heart, indicating the presence of inflammatory cells, we could not detect CD45-/GFP+ cells in the heart of infected chimeric mice, indicating that BM-derived cells do not contribute to heart regeneration during the acute phase of Chagas disease. In contrast, Souza et al. (2014) showed by immunofluorescence analysis that BM cells migrate and contribute to the formation of new resident cells in the heart using a chimeric mouse model. We cannot exclude rare events of CD45-/GFP+ detection (as debris or cells) in our flow cytometry analysis; however, in contrast to immunofluorescence where single events are valued, in flow cytometry, we examined thousands of events. Therefore, we are better able to measure the presence/absence of a cell type using this method.

In conclusion, chimeric mice presented alterations compatible with chagasic cardiomyopathy. There was no migration of CD45low/CD117+ cells and no CD45-/GFP+ cells present in the cardiac tissue. The GFP+ cells that migrated from the BM to the hearts of infected chimeric mice were mainly inflammatory cells.
